# Clinical and imaging factors that can predict contagiousness of pulmonary tuberculosis

**DOI:** 10.1186/s12890-023-02617-y

**Published:** 2023-09-06

**Authors:** Markus Unnewehr, Florian Meyer-Oschatz, Hendrik Friederichs, Wolfram Windisch, Bernhard Schaaf

**Affiliations:** 1 Faculty of Health, Universität Witten/Herdecke, Alfred-Herrhausen-Straße 50, 58448 Witten, Germany; 2 Department of Respiratory Medicine, Infectious Diseases, Sleep Medicine, St. Barbara-Klinik, Am Heessener Wald 1, 59073 Hamm, Germany; 3 Department of Respiratory Medicine, Infectious Diseases, Intensive Care Medicine, Klinikum Dortmund, Münsterstraße 240, 44145 Dortmund, Germany; 4 Department of Anaesthesiology, Emergency Medicine, Intensive Care Medicine, Bundeswehrzentralkrankenhaus Koblenz, Rübenacher Straße 170, 56072 Koblenz, Germany; 5 Faculty of Medicine, Universität Lübeck, Ratzeburger Allee 160, 23562 Lübeck, Germany; 6 Medical Education Research Group, Medical School OWL, Bielefeld University, Universitätsstraße 25, 33615 Bielefeld, Germany; 7 Department of Pneumology, Cologne Merheim Hospital, Kliniken der Stadt Köln gGmbH, Ostmerheimer Str. 200, 51109 Köln, Germany

**Keywords:** Mycobacteria, Prediction, Acid-fast bacilli, Lung infection, Disease, Clinical patient management

## Abstract

**Background:**

Knowledge on predicting pulmonary tuberculosis (PTB) contagiosity in the hospital admission setting is limited. The objective was to assess clinical and radiological criteria to predict PTB contagiosity.

**Methods:**

Retrospective analysis of 7 clinical, 4 chest X-ray (CXR) and 5 computed tomography (CT) signs in 299 PTB patients admitted to an urban tertiary hospital from 2008 to 2016. If the acid fact bacilli stain was positive (AFB+) on admission, the case was considered high contagiosity.

**Results:**

Best predictors for high PTB contagiosity (AFB+) were haemoptysis (OR 4.33), cough (3.00), weight loss (2.96), cavitation in CT (2.75), cavitation in CXR (2.55), tree-in-bud-sign in CT (2.12), German residency of the patient (1.89), and abnormal auscultation findings (1.83). A previous TB infection reduced the risk of contagiosity statistically (0.40). Radiographic infiltrates, miliary picture, and pleural effusion were not helpful in predicting high or low contagiosity. 34% of all patients were clinically asymptomatic (20% of the highly contagious group, 50% of the low contagious group).

**Conclusion:**

Haemoptysis, cough and weight loss as well as cavitation and tree-in-bud sign in CXR/CT can be helpful to predict PTB contagiosity and to improve PTB management.

**Supplementary Information:**

The online version contains supplementary material available at 10.1186/s12890-023-02617-y.

## Introduction

Preventing aerogenous transmission of pulmonary tuberculosis (PTB) in a clinical setting requires enormous logistical, personal and financial resources by isolating the patient until diagnostic is performed [1–3]. Over-protection can lead to a waste of resources [4, 5]. On the other hand, a lack of transmission protection can put fellow patients and hospital personnel at risk [6]. If TB suspicion is not apparent on admission, it takes 3.3 days in average until the patient is isolated. During this time, 41 members of hospital staff are exposed to one patient [7].

Fast and reliable identification and isolation of persons under evaluation, and treatment initiation are, therefore, the mainstays of the prevention of TB transmission. Patients with possible transmittable PTB have to be isolated until contagiosity is ruled out, ideally by negative microbiological parameters [8–12]. The decision to isolate has to be made during the first contact with the admitting physician, in the admissions department, walk-in clinic or emergency department of a hospital or at a doctor’s office. Here, epidemiological, clinical, and radiological features, as well as the medical history, are the only features available, while microbiological (sputum) essays are in processing.

Even though the duration of sputum acid fast bacilli (AFB) staining and microscopy itself only takes a few hours, the whole process can take days, depending on the microbiological laboratory logistics of the medical facility. TB cultures take weeks to finish. There is no reliable point-of-care test for TB, and also PCR testing usually requires several days because of logistic issues.

Only few studies of low quality, compared to today’s requirements, deal with TB transmission [13] and clinical features at all [14–16]. Fever, lung consolidation, positive tuberculin skin test (TST), and TB symptoms can predict a positive TB culture [16, 17]. For industrial countries, there are no studies on clinical and radiological criteria to predict PTB contagiosity.

### Objective

To identify aspects in patients’ history as well as radiological and clinical criteria to predict PTB contagiosity and to prompt the necessity of isolation in the admission setting. The criteria should be reliable and easy to obtain during first patient contact. The prevalence of symptoms and clinical findings, characteristic for PTB, and special clinical features, were to be evaluated.

The following questions were to be addressed:

1. In case of PTB suspicion, which parameters from the patients’ medical history, clinical findings (e.g. from physical examination), and radiological findings should raise suspicion for high contagiosity prompting isolation?
2. What are the conclusions for the clinical management?

## Methods

### Clinical and radiological criteria

Signs and symptoms of PTB were identified by detailed literature search (see Additional file 1) [14, 18–23].

The admission process was analyzed step-by-step, and compared with the literature search results. Pre-existing conditions could not be included because of incompleteness and heterogeneity of the data. After excluding unspecific and rare signs, the following, directly available criteria during the admission process were extracted from the literature and included in the analysis:

### Clinical

Sex, age, weight loss (loss of 10% or more body weight in 10 months or less), cough, haemoptysis, previous TB infection, foreign / German residency (main country of residence in the last years), positive auscultation (any abnormal chest auscultation finding, no further subdivision due to difficulty categorizing and depending on examiner).

### Chest x-ray (CXR)

Infiltrate, cavitation, miliary picture, pleural effusion.

### Computed tomography (CT)

Infiltrate, cavitation, miliary picture, pleural effusion, tree-in-bud sign.

CT was included since this diagnostic tool is widely available in emergency departments and admission units in developed countries. The tree-in-bud sign is a finding which can solely be found in CT images. Fever was not included, since it was suggested to be a rather unspecific clinical sign in the admission setting. Microbiological parameters and additional laboratory findings such as Interferon-gamma-release assays (IGRA), tuberculin-skin-test (TST) and HIV status were not included because of the diagnostic delay.

Case detection by active and passive case finding was also considered (Table 1).

**Table 1:** Criteria analyzed for the purpose of the study

Criteria		Specifications
*General data*		
Sex		Male / female
Age		On admission
*History, signs, symptoms*		
Weight loss		Present / absent As reported by the patient
Cough		
Haemoptysis		
Previous TB infection		
Residency		German / foreign residency in the last years
Case finding		Active / passive
Chest auscultation		Any abnormal / normal findings
*Radiological findings*		
Chest X-ray	Infiltrate	Present / absent Identified by senior radiologist Infiltrate: irrespective of shape, including consolidations Tree-in-bud-sign: only CT findings Others: non-TB findings
	Cavitation	
	Miliary picture	
	Pleural effusion	
	Others	
Chest CT	Infiltrate	
	Cavitation	
	Miliary picture	
	Pleural effusion	
	Tree-in-bud-sign	
	Others	

### Study design

The study was carried out as a retrospective data analysis of all patients diagnosed and treated for PTB in a regional TB center in an urban tertiary care hospital in Germany (Klinikum Dortmund, Department of Respiratory Medicine) between 7/2008 and 12/2016.

### Inclusion criteria

18 years of age on admission, lung parenchyma involvement, PTB diagnosis and treatment according to national and WHO standards.

### Exclusion criteria

**Fig. 1:**
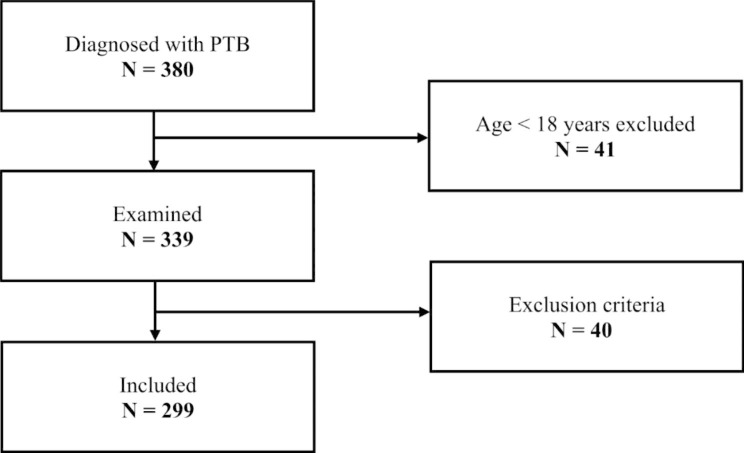
Flow chart of the inclusion and exclusion criteria of the patients

< 18 years of age at admission, diagnostic errors (e.g. uncertain diagnosis), extrapulmonary TB without lung parenchyma involvement, nontuberculous mycobacteria (NTM) infection, admission for treatment evaluation, complications, follow-up of a known TB infection, TB treatment started before admission (Fig. 1).

Data of patients included in the study were extracted from the hospital data base. Each patient file was reviewed in detail by the authors.

During the admission process, the medical history was taken, followed by a clinical examination and a CXR plus a lung CT, if the CXR finding was not considered typical for PTB (e.g. cavity in the upper lobes) by the admitting physician. The radiological examinations were read by qualified consultant radiologists; the findings were documented according to general standards of the hospital. Based on these results, the decision to isolate the patient was made.

### Microbiology

Microbiological specimens were obtained according to usual standards. If expectoration was not possible, inhalation of 3% saline solution and / or bronchoscopy were performed as clinically indicated. Specimens were obtained before initiating antituberculotic treatment. AFB stains were carried out using Kinyoun staining (AB Diagnostics™) and Auramin staining (Merck™). TB cultures were set up by one liquid and two solid cultures (MGIT, BD Diagnostics™, Löwenstein-Jensen (Oxoid™), Stonebrink (Oxoid™)) according to national TB guidelines. The participating hospital laboratories were subject to regular quality controls according to legal requirements.

### Allocation of the patients

All parameters (Table 1) were regarded dichotomously, i.e. “present / absent”, or “yes / no”. The patients were assigned to 2 groups according to the final contagiosity status by microbiological results obtained later:

#### Group 1: Highly contagious (AFB+) patients

According to national and international recommendations [10, 12, 24], these patients with at least one microscopically positive stain (AFB+) in sputum or bronchial lavage during their hospital stay, expectorate a transmissible number of bacteria and are, therefore, considered to be highly contagious, and to be isolated.

#### Group 2: Lowly contagious (AFB-) patients

Patients with negative AFB stain during the hospital stay were considered lowly contagious according to official German standards [10, 25], regardless of the culture result obtained weeks later. On purpose, TB culture und PCR results were ignored since they can not lead to isolation in the admission situation.

### Statistical analysis

Data was analyzed by SPSS™ (IBM™) statistics software using crosstabulation with criteria in Table 1 as dependent and with contagiosity as independent factors. The parameters were analyzed independently of one another to exclude misinterpretations by mutual interactions. Multivariate analysis showed no advantage over univariate.

Hypothesis tests were performed by Chi-square test. The significance level was, as usual, defined as < 0.05 (significant), < 0.01 (very significant) and < 0.001 (highly significant). For very small samples (miliary picture in CXR/CT), Fisher’s exact test was used. The extent of correlation of the criteria were scrutinised by calculating the odds ratio. Sensitivity and specificity of the criteria in terms of the prediction of the contagiosity were calculated as well.

## Results

### General data

299 patients were included in the final analysis, of which 76% (n = 226) were male, 24% (n = 73) were female. The mean age was 40.4 years with a range from 18 to 90 years.

97% (n = 100) of the patients diagnosed by active case finding, were of foreign residency. 151 (50%) were AFB + on admission (group 1), 148 (50%) were AFB- (group 2).

The patients’ medical history, signs and symptoms are displayed in Table 2, the radiological findings in Table 3. CXR was performed in 91% (n = 272) of the patients, lung CT in 93% (n = 279).

**Table 2:** Patient’s history, signs, symptoms

	Present/positive (n)	Absent/negative (n)	No data (n)
Weight loss	34% (103)	61% (182)	5% (14)
Cough	43% (127)	52% (154)	6% (18)
Haemoptysis	10% (31)	86% (257)	4% (11)
Previous TB	24% (72)	65% (193)	11% (34)
German residence	22% (66)	72% (214)	6% (19)
Active case finding	35% (104)	65% (195)	0% (0)
Abnormal auscultation	22% (66)	75% (224)	3% (9)

**Table 3:** Radiological findings

	Present (n)	Absent (n)
*CXR (n = 272)*		
- Infiltrate	85% (230)	15% (42)
- Cavitation	27% (74)	73% (198)
- Miliary picture	2% (5)	98% (267)
- Pleural effusion	11% (29)	89% (243)
- Others^+^	3% (9)	
*CT (n = 279)*		
- Infiltrate	72% (201)	28% (78)
- Cavitation	49% (138)	51% (141)
- Miliary picture	3% (9)	97% (270)
- Pleural effusion	13% (35)	88% (244)
- Tree-in-bud-sign	25% (70)	75% (209)
- Others^+^	2% (5)	

^+^Any pathological finding not mentioned in the list

CXR detected more infiltrates than CT (85% vs. 72%). This can be explained by the lower specificity of CXR, so that other findings were summed up and regarded as infiltrates. CT identified more cavitations, which were most likely pointed out as infiltrate on CXR. Details can be found in Table 4.

**Table 4:** Comparison of the high and low contagious groups

	Overall prevalence	Prevalence in high contagious group (AFB+)	Prevalence in lowly contagious group (AFB-)	p
*History, signs, symptoms*				
Weight loss	36%	48%	24%	0.000***
Cough	45%	58%	31%	0.000***
Haemoptysis	11%	17%	4%	0.001**
Previous TB	27%	19%	37%	0.001**
German residency	24%	64%	36%	0.028*
Passive case finding	65%	58%	42%	n. s.
Active case finding	35%	37%	64%	0.000***
Auscultation	23%	28%	18%	0.035*
No history and findings	34%	20%	50%	0.000***
*Radiological findings*				
*CXR*				
- Infiltrate	85%	83%	87%	0.394
- Cavitation	27%	36%	18%	0.001***
- Miliary picture	2%	2%	2%	1.000
- Pleural effusion	11%	13%	8%	0.211
- Others^+^	3%	3%	5%	0.684
*CT*				
- Infiltrate	72%	70%	74%	0.491
- Cavitation	50%	62%	37%	0.000***
- Miliary picture	3%	4%	2%	0.501
- Pleural effusion	13%	16%	9%	0.119
- Tree-in-bud sign	25%	32%	18%	0.008**
- Others^+^	2%	1%	3%	0.168

Calculation by univariate analyses for each parameter using Pearson’s Chi-square test except Fisher’s exact test for “Miliary picture in CXR” and “Miliary picture in CT”. Prevalence calcuation after excluding no data-cases

* = significant, ** = very significant, *** = highly significant

^+^Any pathological finding not mentioned in the list

As shown in Table 5, best predictors for high TB contagiosity were haemoptysis (OR 4.33), cough (3.00), weight loss (2.96), cavitation in CT (2.75), cavitation in CXR (2.55), tree-in-bud in CT (2.12), non-foreign residency of the patient (1.89), and abnormal auscultation findings (1.83). A previous TB reduced the risk of contagiosity statistically (OR 0.40) (Table 5).

**Table 5:** Statistically significant results from Table 5, by PPV, 95% CI and OR for high contagiosity, and sensitivity and specificity. Assorted by OR

	OR	95% CI	PPV	Sensitivity	Specificity
Haemoptysis	4.33	1,7–10,9	81%	17%	96%
Cough	3.00	1,8 − 4,9	67%	58%	69%
Weight loss	2.96	1,8 − 4,9	67%	48%	76%
Cavitation in CT	2.75	1,7 − 4,4	62%	62%	63%
Cavitation in CXR	2.55	1,5 − 4,5	68%	36%	82%
Tree-in-bud in CT	2.12	1,2–3,7	64%	32%	82%
Non-foreign residency	1.89	1,1–3,3	48%	71%	18%
Auscultation	1.83	1,0–3,2	62%	28%	83%
Active case finding	0.42	0,3 − 0,7	58%	75%	45%
Previous TB	0.40	0,2 − 0,7	36%	19%	64%

### Statistically not significant results

The following criteria were not helpful in identifying highly contagious patients as they revealed no significant difference: Infiltrate in CXR, infiltrate in CT, miliary picture, pleural effusion.

### Other relevant results

34% of all patients were clinically asymptomatic (no haemoptysis, cough, weight loss, auscultation).

20% of the highly contagious patients were asymptomatic, 50% of the lowly contagious group. This is a statistically significant difference (p < .001) (Table 4).

CXR and CT detected abnormal findings in a similar accuracy, while CT showed more TB specific findings than CXR (cavitation, tree-in-bud sign).

## Discussion

This study is the first to provide robust data about PTB contagiosity (AFB+) prediction by haemoptysis, cough, weight loss, abnormal auscultation findings, cavitary lesion on CXR and CT, and tree-in-bud-sign on CT in a developed country.

The general data obtained (e.g. age pattern, sex, residency) are similar to those obtained by the German TB public health survey (male sex 70% vs. 76% (our study), median age 34 years vs. 37 years, non-German residency 65% vs. 72%, AFB + and culture + 43% vs. 51%). A comparison with European countries with comparable socioeconomic structure to Germany (e.g. Sweden, Norway, the Netherlands, Austria) yields a similar result. Therefore, our data seem to be representative for the situation in Germany and in other European countries, even though our study has a monocentric design [9, 26].

The results are compatible with pathophysiology and clinical experience. Severe inflammation and bacterial growth can lead to haemoptysis by vascular involvement. Weight loss is a sign of advanced stage of TB which goes along with increased bacterial expectoration. Cavitary lesions typically have contact to the bronchial system which explains contagiosity, as does cough. A tree-in-bud sign as a sign of bronchioalveolar involvement and marker of disease activity leads to high contagiosity, while a simple infiltrate does not necessarily increase the risk [14, 15, 21]. A miliary TB is a less liable parameter as there were too few cases in our study. Pleural involvement does usually not lead to high contagiosity since the pleural cavity does not have a direct contact to the respiratory system.

The risk reduction of contagiosity, indicated by a previous TB infection can be explained by an acquired protection of the host by the first TB, as shown in animal models [27] and in immunological studies [28, 29]. More probable as an explanation is, however, the raised level of suspicion aroused by the admitting physicians which leads to a diagnosis in an earlier disease stage.

The allocation of the patients to two groups according to their AFB stain status reflects daily clinical practice, where AFB stain plays a crucial role in the early decision process of isolating a patient. AFB- patients during the hospital stay are considered less contagious according to official standards [10, 25], regardless of the culture result obtained weeks later. In clinical practice, patients with low clinical and radiological TB probability (e.g. no CXR/CT findings) can be de-isolated if one AFB is negative [10, 30]. On purpose, TB culture results had to be ignored in the current study, since they were unknown in the admission situation. AFB stain in this context can only be regarded as one marker of “contagiosity” among others.

Two studies stress the uncertainties in TB transmission. In a retrospective study on all Danish TB patients in 5 years, the AFB grading was associated with the relative transmission risk [31]. On the other hand, a recent study in South Africa showed no correlation between AFB grade and bacteria output, as measured by a new face mask sampling method over 24 h, however with a small number of patients and a high dropout rate [32]. This underlines that additional investigation is required regarding TB infectivity, especially sputum AFB positivity.

Literature scrutiny revealed two studies on the prevalence of PTB symptoms, both carried out in Zambia and Kenia [14, 15]. The higher number of patients with weight loss in the African studies can be explained by the higher percentage of HIV/AIDS patients in Africa and the limited access to medical care which explains a diagnosis in an advanced and more severe stage of disease. This also explains the higher percentage of asymptomatic PTB patients in our study. The compulsory TB screening among migrants arriving in Germany also plays a role.

Considering these differences, the results of our study are feasible and allow to draw conclusions for more developed countries.

The interesting result of 34% asymptomatic patients underlines the rationale of current screening, mainly by CXR and laboratory tests (IGRA). History and clinical findings alone are not sufficient to establish or rule out TB [21].

Limitations of our study are the dichotomous characterizations, which were chosen for reasons of feasibility during the hospital admission, of a higher degree of standardization to increase the reliability among the admitting clinicians, and of statistical evaluation. More detailed classifications would have been too difficult to analyze and would not necessarily have increased validity [33]. For the same reasons, a grouping of radiological findings was carried out. A clinical real-life setting was chosen, and the usual course of patient management was followed and analyzed. The retrospective character of this approach might be a limitation of the conclusions.

Including AFB results in the study as part of the reference used, leads to a neglectable incorporation bias [34].

TB PCR was not included in the study since it was not a standard part of the diagnostic process in the first years of the study period. If obtained, however, it was included in the usual diagnostic and therapeutic decision process.

The results have a high impact on the clinical management. Before the admission process, a questionnaire e.g. by telephone including markers for high contagiosity can optimize the pre-clinical process of isolation, transport, and hygiene setting during admission. The higher rate of TB specific radiological findings in CT than in CXR (cavitation, tree-in-bud) should lead clinicians to consider CT more frequently. CT adds to the diagnostic accuracy, and to the prediction of contagiosity. This gain of information has always to be weighed up with the exposition to radiation.

Future studies should deal with direct measuring of expectorated bacteria by aerosol analysis [35] and could include point-of-care laboratory tests. Another promising approach to assess PTB contagiosity is the involvement of artificial intelligence (AI) by analyzing clinical, radiological and epidemiological parameters [36].

## Conclusions

Our study provides valuable information about TB contagiosity. Haemoptysis, cough, weight loss, and abnormal auscultation findings can raise suspicion of contagious TB (AFB+), as well as cavitary lesions in CXR and CT, and a tree-in-bud sign in CT. These findings in the admission setting should prompt initial isolation of the patient.

CXR has a similar accuracy in detecting abnormal findings, while CT is more PTB-specific.

### Electronic supplementary material

Below is the link to the electronic supplementary material.

Additional file 1. Keywords used in the primary literature search.

## Data Availability

The datasets used and/or analysed during the current study are available from the corresponding author on reasonable request.
