# Supplementary material for: Clinical and imaging factors that can predict contagiousness of pulmonary tuberculosis
Source: BMC Pulm Med. 2023 Sep 6;23:328. doi: 10.1186/s12890-023-02617-y (PMC10481505; doi:10.1186/s12890-023-02617-y)
Supplement: Supplementary file 1 — Additional file 1. Keywords used in the primary literature search. [file 12890_2023_2617_MOESM1_ESM.docx]

**Additional file 1**

*Keywords used in the primary literature search*

Cough, acute cough, chronic cough, sputum production, haemoptysis, weight loss, anorexia, fever, night sweats, chills, previous TB, former treatment for TB, family history of TB, shortness of breath, difficulty in breathing, dyspnea, chest pain, pleuritic complaints, fatigue, weakness, pulmonary auscultation, rales, abnormal chest radiograph, upper lobe infiltrate, apical upper lobe involvement, cavern, cavernous lesion, scattered lesions, fibronodular infiltrates, consolidations, miliary tuberculosis, focal bronchopulmonary pneumonitis, tree-in-bud pattern, lymphadenopathy, lymph node enlargement, hilar and mediastinal lymph node enlargements, satellite foci, focal pleural thickening, pulmonary nodules, small nodules, solitary pulmonary nodule, Ghon focus, calcified primary complex, atelectasis, pleural effusion, unilateral pleural effusion, bronchiectasis.
